# The Power of Time: Editorial on the Advantages of Electroencephalography (EEG) and Event-Related Potentials (ERPs) in Affective and Cognitive Neuroscience

**DOI:** 10.3390/brainsci15101054

**Published:** 2025-09-28

**Authors:** Peter Walla

**Affiliations:** 1 Faculty of Psychology, Freud CanBeLab, Sigmund Freud Private University, Freudplatz 1, 1020 Vienna, Austria; peter.walla@sfu.ac.at; 2 Faculty of Medicine, Sigmund Freud Private University, Freudplatz 3, 1020 Vienna, Austria; 3 School of Psychology, Newcastle University, University Drive, Callaghan, NSW 2308, Australia

## 1. Introduction

### 1.1. The Temporal Lens of the Brain

The central argument of this editorial is that EEG and ERPs offer an unparalleled temporal resolution, enabling the dissection of neural events in the millisecond range [1]—a capability that distinguishes them from methods like functional Magnetic Resonance Imaging (fMRI), which, despite superior spatial resolution, are inherently limited by the slower hemodynamic response [2,3]. This temporal precision, coupled with their non-invasiveness, cost-effectiveness, and direct measurement of neural activity, positions EEG and ERPs as critical tools for investigating the dynamic and often non-conscious aspects of brain function [4,5]. The ability of ERPs to capture neural activity with millisecond precision allows researchers to dissect the rapid sequence of cognitive and affective operations from the earliest sensory registration to complex decision-making. This temporal granularity is crucial for understanding the dynamic interplay of neural processes underlying human thought and behavior.

### 1.2. Direct Measure of Neural Activity

Unlike fMRI, which measures blood-oxygen-level-dependent (BOLD) signals—an indirect proxy for neural activity reflecting metabolic changes—EEG directly measures the electrical potentials generated by neuronal populations [6]. This offers a fundamental and more immediate insight into brain function, as electrical signals are the direct language of the brain. All nine papers from the Special Issue “EEG and ERPs”, by their very nature of utilizing EEG/ERPs, implicitly rely on this direct measurement, which is the core principle enabling their precise temporal insights. This directness means that EEG/ERPs are less susceptible to the physiological lags and interpretive ambiguities associated with indirect measures, providing a more immediate reflection of underlying neuronal processes and how neurons communicate and process information.

## 2. The Special Issue on EEG and ERPs Highlights the Versatility Across Various Research Paradigms

The adaptability of EEG/ERPs allows their application across an exceptionally broad range of research questions and experimental designs, from basic sensory processing to complex social interactions and clinical conditions. The sheer diversity of the nine papers serves as the strongest evidence for this versatility.

In this Special Issue, EEG/ERPs have been effectively applied to the following:

- Empathy and Social Cognition: Investigating early perceptual stages of empathic processing and gender differences in face processing.
- Clinical Diagnostics and Treatment Monitoring: Assessing post-COVID-19 concentration disorders, long-term neurophysiological consequences of complex childhood trauma, and cognitive deficits in depression, as well as monitoring therapeutic outcomes.
- Consumer Behavior and Neuromarketing: Uncovering implicit attitudes and the effects of evaluative conditioning on brand perception.
- Human–Computer Interaction and Motor Control: Developing brain–computer interfaces (BCIs) for motor imagery classification and exploring the neural basis of rhythmic sensorimotor synchronization and mental imagery.
- Cognitive Assessment and Expertise: Objectively assessing expert knowledge by revealing distinct neural processing patterns of domain-specific language.

## 3. Details About All Contributions in This Special Issue

In the following, you will find a more detailed summary across all nine contributions, including overarching neuroscience topics grouping all papers.

### 3.1. Capturing the Fleeting Moments of Perception and Attention

Early ERP components provide critical insights into the initial, often automatic, stages of sensory and perceptual processing that occur within milliseconds of stimulus presentation. These components reveal how the brain rapidly filters, prioritizes, and categorizes incoming information. The visual P1 component, typically peaking around 100 ms at occipital electrodes, is an early marker of visual attention and sensory processing [7]. Its modulation can indicate preferential processing of certain stimuli. Research exploring gender differences and opposite-gender biases in face processing has leveraged the P1 component to reveal rapid, attention- and expression-dependent modulations of early visual processing. One study from the current Special Issue found an opposite-gender bias at P1 for happy target faces (contribution 1). Specifically, women exhibited larger P1 amplitudes when viewing male happy target faces compared to female happy target faces, while men showed larger P1 amplitudes for female happy target faces compared to male happy target faces. This effect was notably restricted to target faces and happy expressions, suggesting that top-down attentional templates, possibly influenced by evolutionary mate selection cues, can rapidly shape even the earliest sensory processing. This temporal specificity, occurring approximately 100 ms after stimulus onset, highlights the capacity of ERPs to pinpoint the precise moment when specific cognitive biases emerge, offering a deeper understanding of social perception. Following P1, the N170 component, an occipito-temporal negativity peaking around 170 ms, is a robust marker of holistic face encoding [8]. Its amplitude is reliably modulated by emotional facial expressions, typically showing greater (more negative) amplitudes for emotional faces (e.g., happy or fearful) compared to neutral faces. However, this component has been observed to remain largely uninfluenced by face or participant gender, suggesting its stability across various attentional conditions once a stimulus is identified as a face. The temporal dissociation between the P1 effect (showing gender bias) and the N170 effect (not showing gender bias) is a compelling demonstration of ERPs’ unique ability to distinguish between very early, attention-modulated sensory processing and subsequent, more configural face encoding stages. This fine-grained temporal mapping is essential for building comprehensive models of social cognition.

Beyond face processing, the N170 component also plays a role in the early perceptual stages of empathic processing. A systematic review of Event-Related Potential studies in perceptual tasks related to empathy found that the N170 component, particularly for facial stimuli, presented negative correlations with cognitive and affective empathy scales (contribution 2). This observation suggests that individual differences in empathy are reflected in the automatic, pre-conscious processing of social cues like faces. While other early components, such as the N100, P200, and Early Posterior Negativity (EPN), were modulated by emotional stimuli (showing larger amplitudes for positive and negative stimuli over neutral), they generally did not present significant correlations with empathy scales, with the N200 component being an exception. The involvement of the N170 in empathic processing, even at such an early stage, underscores the capacity of ERPs to reveal the neural underpinnings of complex traits at their earliest perceptual roots, bypassing conscious introspection. This indicates that empathic processing is not solely a high-level cognitive function, but is deeply intertwined with fundamental perceptual mechanisms.

### 3.2. Decoding Higher-Order Cognitive Processes

Later ERP components extend the temporal lens to more complex and conscious cognitive operations, including attention, memory, decision-making, and semantic integration. The P300 component, a positive deflection typically peaking between 250 ms and 500 ms over centro-parietal scalp regions, is widely associated with higher-order cognitive processes such as attention allocation, working memory updating, and stimulus evaluation [9]. Its latency is thought to reflect the time required for stimulus classification and appraisal. In clinical contexts, the P300 has emerged as a valuable biomarker for cognitive deficits and a sensitive indicator of therapeutic efficacy. A study published in the current Special Issue investigating children and adolescents diagnosed with depression revealed significantly prolonged P300 latencies across various brain regions compared to their typically developing peers (contribution 3). This finding objectively indicated cognitive impairments in attention and memory among depressed participants, providing neurophysiological evidence for the subjective cognitive complaints often reported in depression. Following intervention with either Cognitive Behavioral Therapy (CBT) alone or CBT combined with selective serotonin reuptake inhibitors (SSRIs), the depressed group demonstrated significant improvements, evidenced by reduced P300 latencies and faster reaction times, reaching levels comparable to the control group. This remarkable normalization of the P300 latency post-treatment positions ERPs as a powerful tool for objective clinical outcome measurement, offering neurophysiological evidence of brain plasticity in response to therapy and providing a more direct and unbiased assessment of treatment efficacy than self-report measures alone. The P300′s sensitivity to both the presence of cognitive impairment and its normalization after therapy directly links neural changes to behavioral improvements, validating the intervention from a neurophysiological perspective.

Beyond discrete event-related components, quantitative EEG (QEEG) analysis offers a powerful means to characterize intrinsic brain states by quantifying the spectral distribution of EEG power across different frequency bands [10]. This approach can reveal subtle abnormalities in brain oscillatory patterns associated with various neurological and psychiatric conditions. The utility of QEEG as a sensitive diagnostic tool was demonstrated in a further study in the current Special Issue assessing post-COVID-19 concentration disorders in professional pilots (contribution 4). QEEG revealed significantly higher amplitudes of alpha, theta, and beta2 waves, and significantly lower sensorimotor rhythm (SMR) in pilots recovering from COVID-19 compared to healthy controls. These objective changes in brain wave activity were directly associated with the pilots’ self-reported concentration problems, fatigue, stress, and attention deficits. The study emphasized QEEG’s capacity to detect “even very small changes in the functioning of the cerebral cortex” and “minimal changes in brain wave activity”. This ability to quantify subtle shifts in brain oscillatory patterns provides objective neurophysiological correlates for subjective “brain fog” [11] and concentration issues, demonstrating EEG’s utility as a biomarker for neurological sequelae of systemic illnesses and laying a foundation for targeted neurofeedback therapies. The quantitative nature of QEEG allows for precise measurement of brain rhythm amplitudes, providing a direct link to underlying neural states (e.g., elevated beta2 for tension or reduced SMR for attention deficits), thereby enhancing diagnostic precision beyond qualitative EEG interpretation.

The N400 component, a negative-going ERP peaking around 400 ms, is classically associated with semantic processing and the detection of semantic incongruities or violations of expectation [12]. Its modulation can reflect the brain’s effort to integrate incoming information with the existing semantic knowledge. In the context of empathy, the N400 has provided insights into how individuals process social information involving language and theory of mind. Research published in this Special Issue (contribution 2) suggests that an augmented N400 amplitude is associated with higher empathy scores in tasks involving mental states and social language categories. This indicates that higher empathy is linked to distinct neural processing of semantic incongruities or contextual integration in social communication. The N400′s involvement in empathy for mental states and social language suggests that empathic individuals might process social information, including linguistic cues, with a different neural signature related to meaning integration. This points to a deeper, neurophysiological understanding of how empathy influences our interpretation of others’ thoughts and feelings, revealing a neural signature related to meaning integration in social contexts.

One of the most compelling advantages of EEG and ERPs lies in their capacity to access implicit and non-conscious cognitive processes, bypassing the limitations of conscious introspection and self-report. This is particularly valuable in fields where explicit measures may be influenced by “cognitive pollution” [13]—conscious reasoning or social desirability biases. In neuromarketing, for example, understanding true consumer attitudes is paramount. A further study in this Special Issue investigating sonic influence on initially neutral brands demonstrated that while self-reported explicit responses and Implicit Association Test (IAT) measures of brand attitude remained unchanged after evaluative conditioning, EEG data revealed significant sensitivity (contribution 5). Specifically, the Late Positive Potential (LPP), a slow sustained positivity reflecting sustained attention and motivational significance, showed sensitivity to initial brand ratings and conditioning effects of initially neutral brands, particularly negative conditioning, at frontal electrode locations AF3 and AF4. This finding strongly suggests that “the brain knows more than it admits to consciousness and language,” underscoring the necessity of a multidimensional approach, inclusive of neuroscience, to truly understand consumer attitudes. This ability of EEG to detect implicit attitude changes, even when self-report fails, demonstrates that learning and attitude formation occur at a non-conscious neural level, providing a more objective and potentially predictive measure of consumer behavior. The observed “negativity bias,” where negative conditioning appeared more potent, further exemplifies this implicit sensitivity.

Beyond consumer behavior, ERPs offer a novel and objective metric for evaluating expert knowledge, revealing how specialized training fundamentally reshapes the neural processing of domain-specific language. Another study in this Special Issue on neurophysiological correlates of expert knowledge used ERPs to distinguish between participants trained in law (experts) and those who were not (novices) (contribution 6). In law-trained participants, ERPs (around 450 ms post-stimulus at left posterior P7 and left frontal FC5 electrodes) clearly differentiated between law-relevant terms and law-irrelevant terms (e.g., “fake rights” and “filler words”), with the latter two eliciting strikingly similar brain activity. This pattern was entirely absent in law-untrained participants, whose brains processed all five term categories in a strikingly similar manner. This implicit grouping of “fake rights” with “filler words” by expert brains, contrasted with novice brains treating all terms as general language, offers a profound neurophysiological signature of acquired knowledge structures and “intuition-based decision-making.” The finding that expert brains process “fake rights” as irrelevant, similar to “filler words,” indicates a sophisticated, almost automatic, semantic filtering based on their deep domain knowledge. This has significant implications for personnel assessment, training efficacy evaluation, and even understanding the neural basis of “legal thinking,” providing an objective assessment beyond traditional criteria like qualifications and experience.

### 3.3. Beyond the Surface: EEG Rhythms and Brain States

Beyond the time-locked responses captured by ERPs, the continuous oscillatory activity recorded by EEG provides a rich source of information about intrinsic brain states, offering insights into brain function that extend beyond discrete event processing. Quantitative analysis of these brain rhythms can reveal subtle abnormalities and serve as potential biomarkers.

#### 3.3.1. Resting-State Dynamics and Clinical Biomarkers

Different EEG frequency bands are associated with distinct brain states and cognitive functions [14]. For instance, alpha waves (8–12 Hz) are typically linked to relaxed wakefulness and cortical inhibition, while theta waves (4–8 Hz) are often associated with drowsiness, memory processes, and creative thinking. Beta waves (13–30 Hz) are indicative of active thinking, arousal, and concentration, and sensorimotor rhythm (SMR, 12–15 Hz) is related to relaxed motor inhibition and attention. Quantitative EEG (qEEG) analysis allows for the precise measurement of the spectral distribution of these rhythms, providing a stable “neurophenotype” that can be associated with neural system functions. Resting-state EEG, particularly qEEG, offers a non-invasive window into the long-term neurophysiological consequences of early life adversity. A further study in this Special Issue investigating the impact of complex childhood trauma (CCT) in institutionalized adolescents revealed significant alterations in resting-state alpha power (contribution 7). Specifically, temporal–posterior alpha power was significantly lower in adolescents exposed to CCT compared to healthy controls in the eyes-open condition. This finding suggests that childhood trauma exposure may have a measurable impact on alpha oscillatory patterns, potentially indicating a higher cortical activation of temporal–parietal areas in traumatized individuals. The study also observed a “double alpha peak” in the trauma group, which could potentially be interpreted as being generated by two or more independent generators from different brain regions, hinting at dysregulated neural networks. This observed reduction in alpha power in CCT adolescents, which contrasts with some findings in adult PTSD literature, suggests an age-dependent or trauma-specific neural signature, emphasizing EEG’s sensitivity to developmental effects and its potential for early, objective screening for complex trauma. The age-related differences in the power of frequency bands underscore the need for developmental specificity in biomarker research, as alpha power might not be very stable with age. Resting-state EEG provides a baseline measure of brain activity, and the observed alpha power differences offer a potential neurophysiological biomarker, crucial for early identification and guiding interventions like neurofeedback, especially in vulnerable populations where self-report might be unreliable.

#### 3.3.2. Real-Time Brain–Computer Interfaces and Motor Control

EEG’s direct measurement of brain electrical activity makes it a foundational technology for brain–computer interface (BCI) systems, particularly in capturing motor imagery (MI) related signals, such as Event-Related Synchronization (ERS) and Event-Related Desynchronization (ERD) [15]. These signals, reflecting changes in brain oscillations during imagined movements, can be decoded to control external devices.

The practical utility of EEG in applied neuroscience, specifically BCI, is continually enhanced by methodological advancements in signal processing. The high dimensionality of multichannel EEG data can introduce issues such as distortion, excessive computational complexity, and data redundancy, which affect BCI performance. To overcome these limitations, an entropy-based channel selection approach has been developed for multichannel EEG (contribution 8). This method identifies channels with higher information content (higher entropy scores) and discards redundant or noisy channels, leading to reduced computational complexity and improved classification accuracy for motor imagery tasks. The use of Common Spatial Pattern (CSP) for extracting spatial features from sub-bands of EEG signals is highlighted as a key technique for MI classification, demonstrating how computational methods can significantly enhance EEG’s efficiency and accuracy for real-time applications. EEG-based BCI is noted for its ease of implementation and cost-effectiveness compared to other neuroimaging methods like fMRI or MEG. This intelligent channel selection, based on information theory, optimizes EEG data for BCI, making it faster and more accurate, which is a critical step towards making BCIs practical for daily use.

Furthermore, EEG-derived measures are pushing the boundaries of what can be inferred from scalp recordings, extending their reach to deep brain activity and complex cognitive control mechanisms. A final study in this Special Issue on rhythmic sensorimotor synchronization introduced Event-Related Deep Brain Activity (ER-DBA), derived from scalp EEG alpha 2 power, to evaluate the dynamics of the dorsal anterior cingulate cortex (dACC), a deep brain structure critical for cognitive control and decision-making (contribution 9). ER-DBA activation and deactivation were found to reflect strategic choices of motor control modality (proactive vs. reactive) based on contextual mental imagery during finger-tapping paradigms. Additionally, ERP traces, particularly omission responses (reverse ERPs for missing pulses), confirmed that mental imagery was contextual and updated by environmental changes and response-based abductive reasoning. This groundbreaking development of ER-DBA to infer activity in a deep brain structure from scalp recordings represents a significant methodological innovation, offering a non-invasive window into subcortical functions critical for cognitive control, decision-making, and motor planning. The detailed analysis of omission ERPs further provides unique insights into the brain’s predictive coding and imagery updating mechanisms, demonstrating how EEG can unravel the neural basis of complex, dynamic cognitive processes. By linking scalp EEG signals to dACC activity and specific motor control strategies, this approach provides a comprehensive, temporally precise understanding of the brain’s “internal models” and their dynamic updating, a level of detail crucial for understanding complex behaviors like music performance or skilled motor actions.

## 4. Limitations, Challenges, and Future Directions

While the profound advantages of EEG and ERPs are evident, a comprehensive understanding of their role in neuroscience necessitates acknowledging inherent challenges and outlining pathways for future advancement. A primary challenge for EEG/ERPs is their inherent limitation in spatial resolution. Localizing the exact neural sources of scalp-recorded EEG signals, often referred to as the “inverse problem” [16], remains a complex computational challenge. Furthermore, EEG signals are susceptible to various artifacts, including eye blinks, muscle movements, and environmental electrical noise (e.g., line noise). These artifacts necessitate rigorous preprocessing pipelines, which can be complex and time-consuming. The overall signal-to-noise ratio of EEG, where brain signals are relatively small compared to noise sources, can impact data quality and the clarity of findings.

### 4.1. The Importance of Methodological Standardization and Open Science

A significant impediment to the cumulative progress of EEG/ERP research is the pervasive methodological heterogeneity across studies. This includes wide variability in task design, ERP component definitions (e.g., inconsistent time windows and electrode sites for measuring the same component), and diverse signal processing and data analysis procedures. Such heterogeneity, while reflecting the versatility of the techniques, renders direct comparisons and meta-analyses difficult to perform and interpret. Adhering to recommended guidelines for filtering settings and signal cleaning can further lessen these issues. Beyond methodological consistency, the promotion of open science practices, particularly increased data sharing, is vital. The lack of access to raw EEG data, often due to manufacturer restrictions or proprietary formats, and missing dataset information in published studies, hinders replicability and limits the potential for large-scale meta-analyses. Promoting open data and sharing preprocessing pipelines would significantly enhance scientific validation and accelerate progress in the field.

### 4.2. Emerging Trends and Future Frontiers

Despite these challenges, the field of EEG/ERP research is continuously evolving, driven by technological advancements and innovative computational approaches. The growing synergy between EEG/ERPs and machine learning/artificial intelligence (AI) is a prominent trend. The development of mobile and wearable EEG systems represents another exciting frontier. Miniaturized, portable EEG devices enable real-world, ecological studies outside traditional laboratory settings. This can bridge the gap between lab findings and real-life applications, providing invaluable insights into brain function in naturalistic environments and expanding the scope of research questions that can be addressed.

Furthermore, EEG provides the real-time feedback necessary for neurofeedback, allowing individuals to self-regulate specific brain activities for therapeutic benefit. This personalized approach holds promise for more effective and targeted treatments for a range of neurological and psychiatric conditions. Further evolutionary developments resulted in the emergence of a social lifestyle with

## 5. Conclusions: The Enduring Legacy of EEG and ERPs

EEG and ERPs, despite their inherent limitations in spatial resolution and susceptibility to artifacts, remain indispensable tools for dissecting the complexities of the human brain. Their unique temporal precision, allowing the capture of neural events in the millisecond range, provides an unparalleled window into the dynamic and sequential nature of cognitive and affective processes. This capability is complemented by their non-invasiveness, cost-effectiveness, and direct measurement of neuronal electrical activity, making them accessible and ethically viable for diverse populations, including children and clinical cohorts.

Crucially, EEG and ERPs offer profound access to non-conscious processes, revealing underlying attitudes, knowledge structures, and cognitive biases that may not be accessible through conscious self-report. This has transformative implications for fields ranging from neuromarketing and social psychology to the objective assessment of expertise. The versatility of these measures is evidenced by their successful application across a broad spectrum of research paradigms, from fundamental sensory processing and social cognition to clinical diagnostics, therapeutic monitoring, and advanced brain–computer interfaces. The ongoing evolution of EEG/ERP methodologies, particularly through integration with advanced computational techniques like machine learning and the development of novel inferential measures like ER-DBA, promises to further unlock deeper mysteries of the brain. While challenges related to methodological standardization and data sharing persist, addressing these issues will only strengthen the foundation for future discoveries. The enduring legacy of EEG/ERPs lies in their profound and continuing impact on our understanding of human brain function, fostering innovation in both basic neuroscience and clinical applications, ultimately contributing to improved human well-being.
